# Virtual screening of flavonoids as potential RIPK1 inhibitors for neurodegeneration therapy

**DOI:** 10.7717/peerj.16762

**Published:** 2024-01-22

**Authors:** Asim Kumar Bepari, Swakkhar Shatabda, Hasan Mahmud Reza

**Affiliations:** 1 Department of Pharmaceutical Sciences, North South University, Dhaka, Dhaka, Bangladesh; 2 Department of Computer Science and Engineering, United International University, Dhaka, Dhaka, Bangladesh

**Keywords:** Bioinformatics, Drug discovery, Docking, Molecular dynamics, Simulation, Kinase, Gromacs, AutoDock vina, Computational, Flavonoid

## Abstract

**Background:**

Global prevalence of neurodegenerative diseases such as Alzheimer’s disease and Parkinson’s disease is increasing gradually, whereas approvals of successful therapeutics for central nervous system disorders are inadequate. Accumulating evidence suggests pivotal roles of the receptor-interacting serine/threonine-protein kinase 1 (RIPK1) in modulating neuroinflammation and necroptosis. Discoveries of potent small molecule inhibitors for RIPK1 with favorable pharmacokinetic properties could thus address the unmet medical needs in treating neurodegeneration.

**Methods:**

In a structure-based virtual screening, we performed site-specific molecular docking of 4,858 flavonoids against the kinase domain of RIPK1 using AutoDock Vina. We predicted physicochemical descriptors of the top ligands using the SwissADME webserver. Binding interactions of the best ligands and the reference ligand L8D were validated using replicated 500-ns Gromacs molecular dynamics simulations and free energy calculations.

**Results:**

From Vina docking, we shortlisted the top 20 flavonoids with the highest binding affinities, ranging from −11.7 to −10.6 kcal/mol. Pharmacokinetic profiling narrowed down the list to three orally bioavailable and blood-brain-barrier penetrant flavonoids: Nitiducarpin, Pinocembrin 7-O-benzoate, and Paratocarpin J. Next, trajectories of molecular dynamics simulations of the top protein-ligand complexes were analyzed for binding interactions. The root-mean-square deviation (RMSD) was 1.191 Å (±0.498 Å), 1.725 Å (±0.828 Å), 1.923 Å (±0.942 Å), 0.972 Å (±0.155 Å) for Nitiducarpin, Pinocembrin 7-O-benzoate, Paratocarpin J, and L8D, respectively. The radius of gyration (Rg) was 2.034 nm (±0.015 nm), 2.0.39 nm (± 0.025 nm), 2.053 nm (±0.021 nm), 2.037 nm (±0.016 nm) for Nitiducarpin, Pinocembrin 7-O-benzoate, Paratocarpin J, and L8D, respectively. The solvent accessible surface area (SASA) was 159.477 nm^2^ (±3.021 nm^2^), 159.661 nm^2^ (± 3.707 nm^2^), 160.755 nm^2^ (±4.252 nm^2^), 156.630 nm^2^ (±3.521 nm^2^), for Nitiducarpin, Pinocembrin 7-O-benzoate, Paratocarpin J, and L8D complexes, respectively. Therefore, lower RMSD, Rg, and SASA values demonstrated that Nitiducarpin formed the most stable complex with the target protein among the best three ligands. Finally, 2D protein-ligand interaction analysis revealed persistent hydrophobic interactions of Nitiducarpin with the critical residues of RIPK1, including the catalytic triads and the activation loop residues, implicated in the kinase activity and ligand binding.

**Conclusion:**

Our target-based virtual screening identified three flavonoids as strong RIPK1 inhibitors, with Nitiducarpin exhibiting the most potent inhibitory potential. Future *in vitro* and *in vivo* studies with these ligands could offer new hope for developing effective therapeutics and improving the quality of life for individuals affected by neurodegeneration.

## Introduction

Flavonoids, a large group of polyphenolic metabolites widely distributed in plants, possess diverse bioactivities ranging from antioxidant and anti-inflammatory effects to anticancer and neuroprotective properties, making them potential candidates for therapeutic interventions. Chemically, flavonoids are three-ring 15-carbon compounds sharing a core 2-phenylbenzopyranone skeleton. Depending on the degree of unsaturation and substitutions, they form distinct subclasses, such as flavones, flavonols, flavanones, flavanols, anthocyanidins, and isoflavones. They can be found in various plant-based sources, including fruits, vegetables, grains, and beverages like tea and wine. This computational study focused on screening flavonoids to inhibit the receptor-interacting serine/threonine-protein kinase 1 (RIPK1) to combat neurodegenerative disorders.

Dietary consumption of flavonoids can offer various health benefits, including reduced risk of cardiovascular diseases, cancer, and neurodegenerative disorders (Yao et al., 2004; Kozlowska & Szostak-Wegierek, 2014). Mounting evidence from *in vitro* and animal studies reveals that flavonoids provide remarkable defense from free radicals through scavenging reactive oxygen species (ROS) and reactive nitrogen species (RNS) and upregulating endogenous antioxidants, such as catalase (CAT), glutathione (GTH), and superoxide dismutase (SOD) (Yao et al., 2004; Shen et al., 2022). Flavonoids modulate various inflammatory pathways, including inhibition of pro-inflammatory enzymes like cyclooxygenases (COXs) and lipoxygenases (LOXs) and suppression of inflammatory mediators like tumor necrosis factor-α (TNF-α) and interleukins (ILs) (Gomes et al., 2008).

Neurodegenerative disorders, such as Alzheimer’s disease (AD), Parkinson’s disease (PD), and Huntington’s disease (HD), are characterized by progressive loss of neurons and synapses in the central nervous system (CNS). In recent years, growing evidence has implicated chronic inflammation as a crucial component in the pathogenesis of these diseases. Given the involvement of inflammation in disease progression, targeting the inflammatory pathways has emerged as a potential therapeutic intervention in neurodegenerative disorders.

Flavonoid-rich plant extracts and isolated flavonoids, such as catechins, hesperidin, kaempferol, luteolin, myricetin, naringenin, quercetin, and rutin have been characterized extensively for their biochemical properties and pharmacological activities (Magalingam, Radhakrishnan & Haleagrahara, 2015; Minocha et al., 2022; Calderaro et al., 2022; Bellavite, 2023). In a β-amyloid (Aβ)-induced rat model of AD, oral naringin (100 mg/kg/day) improved object recognition, avoidance, and spatial memory (Choi et al., 2023). Choi et al. (2023) proposed that naringin may play a role in mitigating the imbalance in the hippocampal brain-derived neurotrophic factor (BDNF) signaling. Quercetin-loaded nanoparticles exhibited antioxidant and antiapoptotic effects in a 2021 study where AD was induced in Wister rats by oral administration of AlCl_3_ for 6 weeks (Amanzadeh Jajin et al., 2021). The enriched nanoparticles nullified the AlCl_3_-induced behavioral deficits, as shown in the Morris water maze and a fear-induced passive avoidance test. Quantitative real-time PCR (qRT-PCR) revealed that quercetin nanoparticles prevented mRNA upregulation of *App*, *Nos2* (also known as *iNOS*) and downregulation of *Bcl2* and *Cat* genes in the hippocampus (Amanzadeh Jajin et al., 2021).

Bai et al. (2023) investigated the anti-inflammatory effects of the flavonoid isoliquiritigenin in a mouse model of PD where inflammation was induced by microglia activation following *in vivo* 1-methyl-4-phenylpyridinium stimulation. This study suggested that isoliquiritigenin counteracted the rise in NOS2 and COX-2 in the mouse substantia nigra and lessened PD phenotypes (Bai et al., 2023). Liu et al. (2023) demonstrated that baicalein effectively decreased ROS-induced oxidative stress in rotenone-treated human neuroblastoma cells. Additionally, baicalein prevented lipid peroxidation in the brain and improved motor coordination in a rotenone-induced rat model of PD (Liu et al., 2023).

RIPKs comprise seven members (RIPK1-RIPK7) of the tyrosine kinase-like (TKL) Ser/Thr protein kinase family. The human RIPK1 is a 76-kDa protein (UniProt ID: Q13546) first characterized by Stanger et al. (1995). It has been extensively studied in the context of necroptosis, a programmed form of necrosis triggered by death-domain receptors (DRs) and executed in the absence of caspase activity. RIPK1 inhibition has emerged as a prime target in various neurodegenerative disorders, including AD, PD, and HD (Degterev, Ofengeim & Yuan, 2019; Li & Yuan, 2023; Xu, Zhang & Zhuang, 2023).

This study aimed to explore the interactions between flavonoids and RIPK1 through computational methods to identify CNS-acting RIPK1 inhibitors that could offer neuroprotective effects. We initiated the screening process by performing molecular docking, a widely used computational technique, to predict the binding affinities and interactions between the flavonoids and the active site of RIPK1. To further validate the docking results and to obtain a more accurate representation of the protein-ligand interactions, we carried out replicated molecular dynamics (MD) simulations.

## Materials and Methods

### Ligand library

We downloaded a flavonoid ligand library of 4,858 compounds previously prepared by Jiménez-Avalos et al. (2021) (File S1). Using the Open Babel version 3.1.1, we created sdf files from SMILES (O’Boyle et al., 2011). Next, pdbqt files with 3D coordinates were generated in a multithreaded parallelized ligand preparation pipeline (Samdani & Vetrivel, 2018) leveraging Open Babel (O’Boyle et al., 2011) and GNU parallel (Tange, 2018). Here, the MMFF94 forcefield was used and energy minimization was done with the steepest descent algorithm.

### Protein 3D structure

We downloaded a 3D structure of the human RIPK1 from the RCSB Protein Data Bank (PDB ID: 6NYH). In a previous study, we used this pdb file for molecular docking (Bepari et al., 2022). The x-ray crystal structure has a resolution of 2.10 Å. However, there are many unmodelled residues and several partially modeled residues. Therefore, we generated a template-based homology model of the RIPK1 kinase domain through the SWISS-MODEL webserver (https://swissmodel.expasy.org/) (Waterhouse et al., 2018). Here, we used the target sequence retrieved from UniProt (https://www.uniprot.org/uniprotkb/Q13546/entry#sequences), and 6NYH was fed as the template. Next, we refined the pdb file for docking in UCSF Chimera version 1.17.1 by removing the ligand and adding hydrogen atoms and charges. Finally, a pdbqt file of the macromolecule was generated using the AutoDockTools (Morris et al., 2009).

### Molecular docking

We adopted the docking protocols from a previous study (Bepari et al., 2022). We utilized a 26*26*26 Å gridbox, centered at the XYZ coordinates −17.094, 13.895, and −37.761, corresponding to the center of the reference ligand L8D in 6NYH. We employed AutoDock Vina (Trott & Olson, 2010) for molecular docking, which was parallelized using POAP (Samdani & Vetrivel, 2018). The number of individual runs (exhaustiveness) for each docking simulation was eight. Following the virtual screening, POAP provided us with a ranked list of ligands based on their binding energy (kcal/mol). The pdb files of the top complexes were used for further analysis and MD simulations.

### Pharmacokinetic screening

We predicted the pharmacokinetic parameters of the top 20 compounds using the SwissADME website (Daina, Michielin & Zoete, 2017). A list of canonical SMILES was provided as the input, and the prediction results were downloaded as a CSV file.

### Molecular dynamics simulations

For MD simulations, we used the RIPK1 complexes with the top hits from docking simulations to generate input files through CHARMM-GUI (Jo et al., 2008). We initiated the solution builder tool (Jo et al., 2008) to prepare the protein solution system from the pdb file of the complex. For the ligand, the hetero chain residues were read from a mol2 file previously prepared using Open Babel (O’Boyle et al., 2011), and CHARMM processes were invoked. We adopted the default parameters with some minor modifications. Briefly, an octahedral waterbox with an edge distance of 10 Å was specified to fit the protein. Forty-seven potassium (K^+^) and 40 chloride (Cl^−^) ions were placed using the Monte-Carlo method to neutralize the system. The number of solvent molecules ranged from 14,203 to 14,238.

Energy minimization was set to perform a maximum of 50,000 steps with the steepest descent algorithm. Before proceeding, the system’s maximum force (Fmax) needed to be below 1,000 kJ mol^−1^ nm^−2^. The system was then equilibrated for 100 ps at 300 K using the V-rescale thermostat (NVT: constant number of particles, volume, and temperature). Subsequent simulation steps were run with the leap-frog integrator. Applying the lincs constraint algorithm, the force constants for backbone atoms (BB) and side-chain atoms (SC) were set to 400.0 and 40.0 kJ/mol/nm^2, respectively. Placing the same constraints, 100-ps NPT equilibration was performed at 300 K and one bar using the V-rescale thermostat and the C-rescale pressure coupling (NPT: constant number of particles, pressure, and temperature). The equilibrated system was then subjected to the 500-ns production MD runs (two replicates for each system).

We mostly used the gmx analysis tools of Gromacs to analyze the trajectories. For root-mean-square deviation (RMSD) and root-mean-square fluctuation (RMSF) calculations, we used reference structures drawn from average structures from the trajectories spanning 200 to 500 ns and fitted to the protein backbone. To determine clusters of conformation, we used the gmx cluster tool with an RMSD cutoff of 0.15 nm. Clustering was done for the last 400 ns, and every 500th frame was analyzed. We computed the solvent accessible surface area (SASA) using the gmx sasa tool. For plotting RMSD, the radius of gyration (Rg), and SASA over time, we used the simple moving average of 100 data points.

We have uploaded the topology files of the best ligand for both replicates as raw data (File S2). The initial and final pdb structures for the complexes of the best ligands are available as raw data (File S3). The trajectories for both replicates are available in Figshare (DOI: 10.6084/m9.figshare.24455332).

### Binding free energy

To calculate binding free energies for ligands, we employed the Molecular mechanics/Poisson–Boltzmann surface area (MMPBSA) method (Miller et al., 2012) using gmx_MMPBSA v1.5.0 (Valdés-Tresanco et al., 2021). We extracted 1,000 frames from the last ten ns of the trajectory for the MMPBSA calculations. The binding free energy of the ligand (ΔTOTAL) was calculated by subtracting the free energies of the receptor and ligand from the complex. For each system, the total free energy was the sum of the gas phase energy (GGAS) and the solvation energy (GSOLV). GGAS was contributed mainly from van der Waals interactions (VDWAALS) and electrostatic interactions (EEL), and GSOLV was the sum of polar (EPB) and nonpolar (ENPOLAR) interactions (Miller et al., 2012).

### Data visualization

Data visualization and statistical analysis were performed using open-source Python packages such as pandas, matplotlib, seaborn, and pingouin in a Jupyter Notebook (Kluyver et al., 2016).

## Results

### Homology modeling of RIPK1

**Figure 1:**
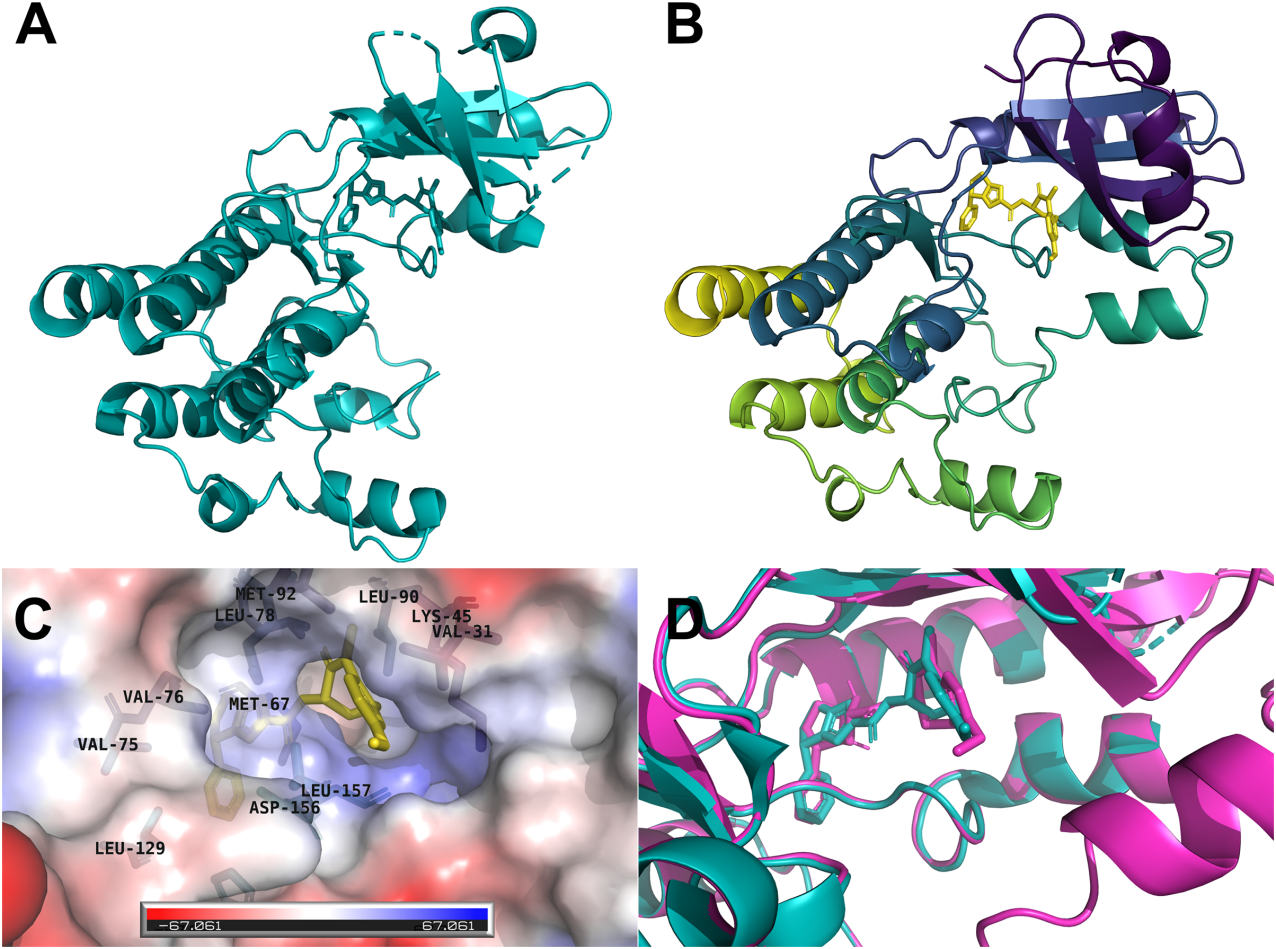
Three-dimensional structures of the human RIPK1. (A) X-ray crystallographic structure 6NYH. (B) RIPK1 kinase domain homology structure. (C) The co-crystallized ligand L8D is buried in a hydrophobic pocket of RIPK1. (D) Redocking of the reference ligand L8D (Magenta: Docking pose, Teal: 6NYH Crystal).

The human RIPK1 protein has 671 amino acids and a mass of 75,931 Da, where the kinase domain resides at the N-terminal (amino acids position 17–289). We selected the x-ray crystallographic structure 6NYH (Fig. 1A) as the template for homology modeling. The 3D structure of the kinase domain we obtained from SWISS-MODEL spanned the amino acid range 8–294, and the missing amino acid residues of the template were successfully modeled (Fig. 1B). The co-crystallized ligand L8D, a potent RIPK1 inhibitor, served as a reference for our docking and molecular dynamics simulations. The inhibitor L8D was buried in a hydrophobic pocket of RIPK1 (Fig. 1C) (Xie et al., 2013).

### Virtual screening of flavonoids by molecular docking

We first redocked the reference ligand L8D to the RIPK1 homology model. The docking pose revealed that our AutoDock Vina docking protocol can reliably imitate the binding conformation of the inhibitor observed in the crystal structure 6NYH (Fig. 1D).

Next, we moved to the initial screening of the flavonoid ligand library with docking simulations. The curated library consisted of 4,858 compounds: 119 from the CHEMBL (https://www.ebi.ac.uk/chembl/), four from the DrugBank (https://go.drugbank.com), and the remaining 4,735 from the Metabolomics (http://metabolomics.jp/wiki/Main_Page) database. For Vina docking, we limited the grid box to the L8D binding pocket (Fig. 1C), which is also the binding pocket for necrostatins, a group of compounds that prevent necroptosis mainly through RIPK1 inhibition (Xie et al., 2013).

We short-listed the top 20 compounds based on the Vina docking scores (kcal/mol), with more negative values indicating stronger binding (Table 1). Scores ranged from −11.7 to −10.6 kcal/mol, and molecular weights ranged from 360 to 724. All the top hits are available in the Metabolomics database: five anthochlors (FL1), four flavanones (FL2), five flavones (FL3), two dihydroflavonols (FL4), two flavonols (FL5), and two isoflavonoids (FLI).

**Table 1:** Top 20 flavonoids from docking simulations.

Name	Metabolomics.JP ID	Docking score (kcal/mol)	MW (g/mol)	Formula
Glyinflanin D	FL1CHYNP0005	−11.7	404.46	C_25_H_24_O_5_
Apigenin 7-glucoside-4′-p-coumarate	FL3FAAGN0001	−11.6	578.52	C_30_H_26_O_12_
Sanggenol C	FL4DAANI0009	−11.1	492.6	C_30_H_36_O_6_
Kanzonol Z	FL4D1ANP0001	−11	406.47	C_25_H_26_O_5_
Biondnoid A, Buddlenoid A, Kaempferol 7- (6′′-p-coumarylglucoside)	FL5FAAGS0060	−11	594.52	C_30_H_26_O_13_
Brosimone G	FL3FALNP0003	−10.9	420.45	C_25_H_24_O_6_
Nitiducarpin	FLID1CNP0005	−10.9	418.48	C_26_H_26_O_5_
Gemichalcone B	FL1C1ANI0027	−10.8	486.51	C_29_H_26_O_7_
Glycyrdione B	FL1CHYNP0009	−10.8	406.47	C_25_H_26_O_5_
Pinocembrin 7-O-benzoate	FL2FA9NS0008	−10.8	360.36	C_22_H_16_O_5_
Paratocarpin J	FL2FAANP0013	−10.8	408.49	C_25_H_28_O_5_
8-Hydroxyapigenin 8- (6″-E-p-coumaroylglucoside)	FL3FFAGS0015	−10.7	594.52	C_30_H_26_O_13_
Puerarol	FLIE1ANI0005	−10.7	404.46	C_25_H_24_O_5_
Sophoradochromene	FL1C1ANP0006	−10.6	458.59	C_30_H_34_O_4_
Demethoxyisogemichalcone C	FL1C1LNI0004	−10.6	502.51	C_29_H_26_O_8_
Euchrenone a2	FL2FAANP0005	−10.6	406.47	C_25_H_26_O_5_
Cycloaltilisin 7	FL2FAANP0012	−10.6	406.47	C_25_H_26_O_5_
Apigenin 7-rhamnoside-4′-rutinoside	FL3FAAGS0033	−10.6	724.66	C_33_H_40_O_18_
Epimedokoreanin A	FL3FACND0001	−10.6	452.45	C_25_H_24_O_8_
(-) -8- (2-Carboxy-1-phenylethyl) -3,5,7-trihydroxyflavone delta-lactone	FL5FA9NN0001	−10.6	400.38	C_24_H_16_O_6_

### Pharmacokinetic evaluation of top flavonoids

**Figure 2:**
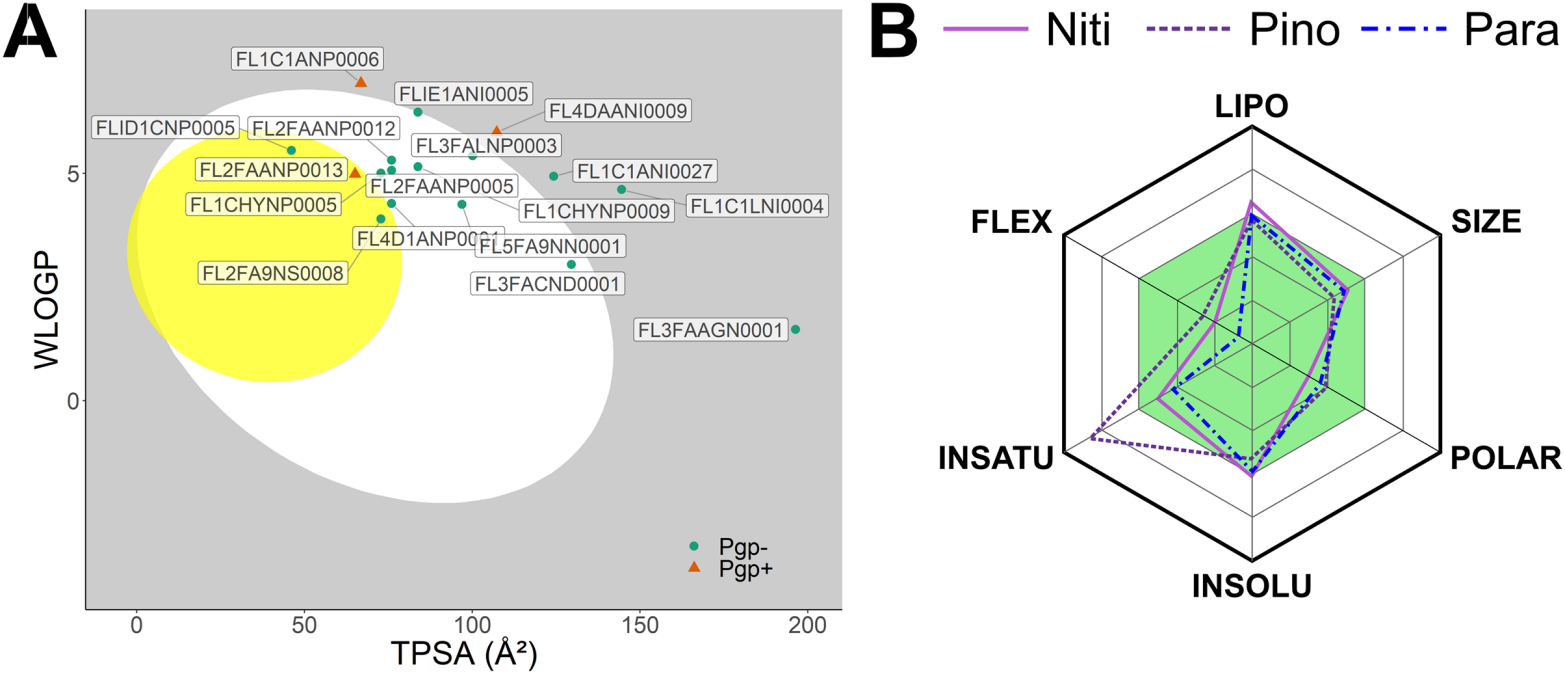
Predicted pharmacokinetic parameters of the top ligands. (A) The BOILED-Egg model showing bioavailability of the top 20 ligands. (B) Radar chart depicting the bioavailability physicochemical space of the top three ligands.

We predicted physicochemical descriptors and pharmacokinetic properties of the top 20 hits from molecular docking (Table S1). Oral bioavailability and blood-brain-barrier permeation are illustrated in Fig. 2A using the BOILED-Egg model (Daina & Zoete, 2016). Twelve molecules showed optimal parameters for gastrointestinal absorption. In contrast, only three molecules, Nitiducarpin (ID: FLID1CNP0005, PubChem CID: 630656), Pinocembrin 7-O-benzoate (ID: FL2FA9NS0008, PubChem CID: 42607888), and Paratocarpin J (ID: FL2FAANP0013, PubChem CID: 15200532), were candidates for blood-brain-barrier penetration. P-glycoprotein (Pgp) transporters translocate substrate out of the cell and can compromise drug actions. SwissADME (Daina, Michielin & Zoete, 2017) revealed that Paratocarpin J is a Pgp substrate while Nitiducarpin and Pinocembrin 7-O-benzoate are not. In the SwissADME output, the physicochemical space for oral bioavailability is further illustrated in radar charts (Fig. 2B), which consolidated six molecular descriptors: lipophilicity (LIPO) as a function of Log *P*_o/w_ (XLOGP3), molecular weight (SIZE), polarity (POLAR) as a function of topological polar surface area (TPSA), insolubility (INSOLU) as a function of Log *S* (ESOL), insaturation (INSATU) as a function of the fraction of sp3 hybridized carbon atoms (Fraction Csp3), and flexibility (FLEX) as a function of the number of rotatable bonds. The radar charts revealed that the three best hits satisfied nearly all the criteria for the above parameters except that Pinocembrin 7-O-benzoate has a higher insaturation than allowed (Fig. 2, Table S1). At this stage, we considered these compounds, Nitiducarpin, Pinocembrin 7-O-benzoate, and Paratocarpin J, the best ligands targeting the human RIPK1 protein.

### Molecular dynamics simulations for top ligands

To validate RIPK1 interactions with the top three ligands, we performed replicated 500-ns MD simulations using Gromacs. As described previously, the co-crystallized ligand L8D in 6NYH was a positive control for the inhibition of RIPK1 (Bepari et al., 2022). RIPK1 complexes with the ligands were placed in an octahedral box, which we solvated with water and then neutralized the system with KCl. Using the steepest descent algorithm, we energy-minimized the protein-ligand complexes (Fig. S1). For the RIPK1-L8D complex, the steepest descents converged to Fmax < 1,000 within 264 steps. The complex with Nitiducarpin also converged very quickly, within 316 steps. The Pinocembrin 7-O-benzoate and Paratocarpin J complexes took 1,004 and 299 steps, respectively, for energy minimization (Fig. S1). Once energy minimization succeeded, the protein complexes in solutions underwent sequential equilibration simulations using NVT and NPT ensembles (Fig. S1). With negligible variations, the MD systems equilibrated at the reference temperature of 300 K and the reference pressure of 1 bar within 100 ps (Fig. S1).

**Figure 3:**
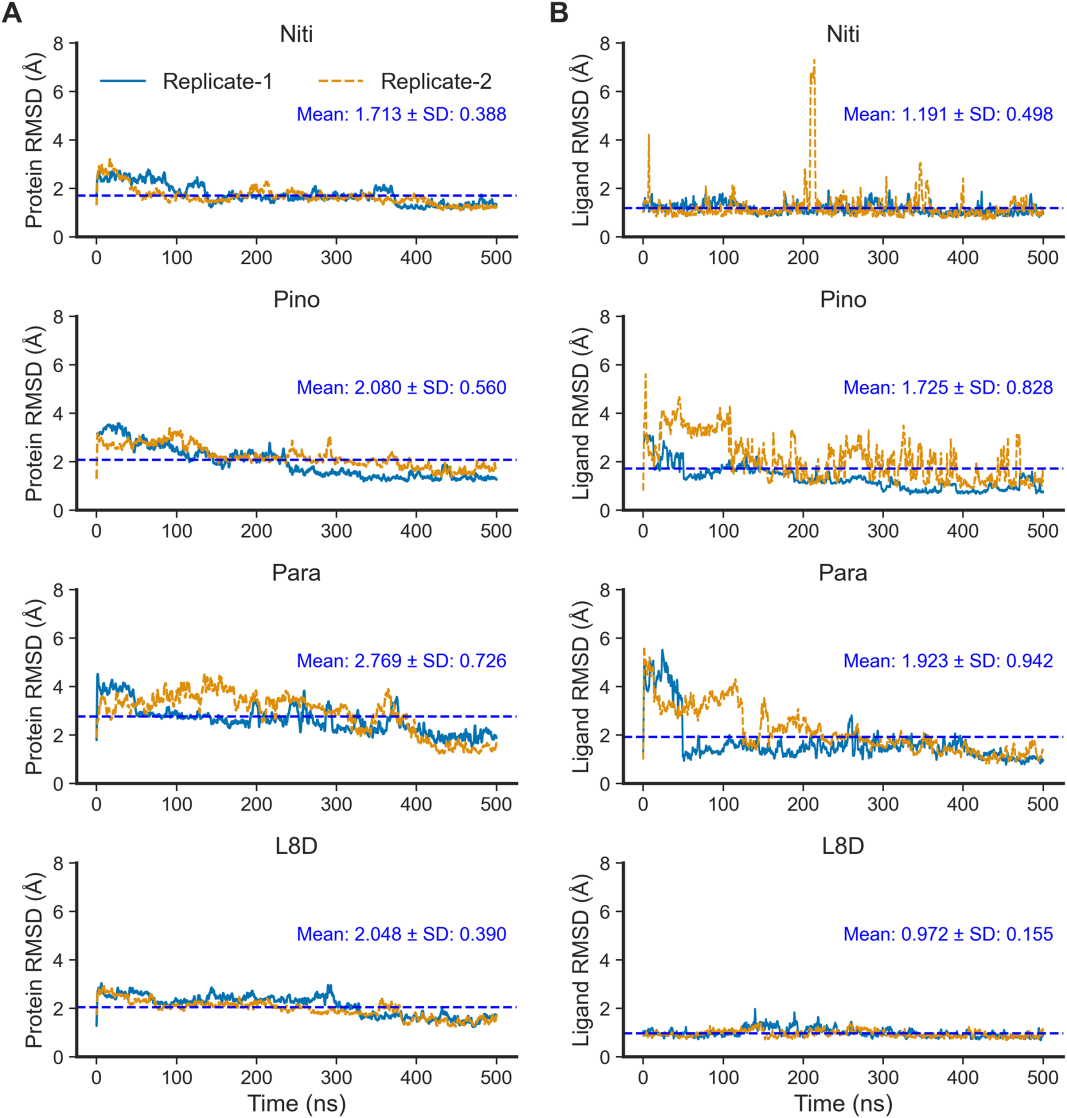
Structural fluctuation of the complexes. (A) Protein backbone RMSD. (B) Ligand RMSD (relative to the protein backbone).

Next, we performed 500-ns production MD runs at NPT. Afterward, the simulation trajectories were analyzed for the stability of the RIPK1 complexes and protein-ligand interactions. The RMSD of the protein backbone atoms (Fig. 2A) is a powerful predictor of protein stability. A low and stable RMSD indicates stable protein conformations within the complex, while a high RMSD points to conformational instability. The reference ligand L8D (also known as GNE684) is a potent pharmacological inhibitor of human RIPK1 *in vitro* (Patel et al., 2020). A low RMSD of the protein backbone in the RIPK1-L8D complex (Fig. 3A) thus strongly aligned with the experimental data (Patel et al., 2020) and validated our MD simulation protocol. The mean protein RMSD was 2.048 Å for L8D, and the RMSD converged very quickly (Fig. 3A). Interestingly, the RMSD for the Nitiducarpin complex converged within the first 100 ns and had the lowest value (1.713 ± 0.388 Å) among the studied complexes (Fig. 3A).

The protein backbone in the Pinocembrin 7-O-benzoate complex also showed considerable stability with a mean RMSD of 2.080 Å. On the other hand, the backbone RMSD was the highest in the Paratocarpin J complex, and we observed convergence only after 400 ns (Fig. 2A).

We next calculated the ligand RMSD (Fig. 3B), a robust metric for validating ligand binding stability following molecular docking simulations (Guterres & Im, 2020). Again, the reference ligand L8D exhibited minimal fluctuations from the protein backbone, with a mean RMSD of 0.972 Å (Fig. 3B), indicating the stability of the ligand within the binding pocket. Nitiducarpin briefly exited the binding cavity at around 210 ns in one of the replicates (Replicate-2) (Fig. 3B). Nonetheless, Nitiducarpin had the lowest ligand RMSD (1.191 ± 0.498 Å) and achieved the fastest convergence among the three hits in our study (Fig. 2B).

Although Pinocembrin 7-O-benzoate exhibited low fluctuations in one simulation, the RMSD varied considerably in the replicated simulation. Paratocarpin J displayed higher structural fluctuations early in the MD simulations. The RMSD converged below the mean at 50 ns in Replicate-1 and at 280 ns in Replicate-2 (Fig. 3A). Taken together, the protein backbone and the ligand RMSD values convincingly demonstrate the stable binding of Nitiducarpin in the inhibitor-binding cavity of RIPK1.

**Figure 4:**
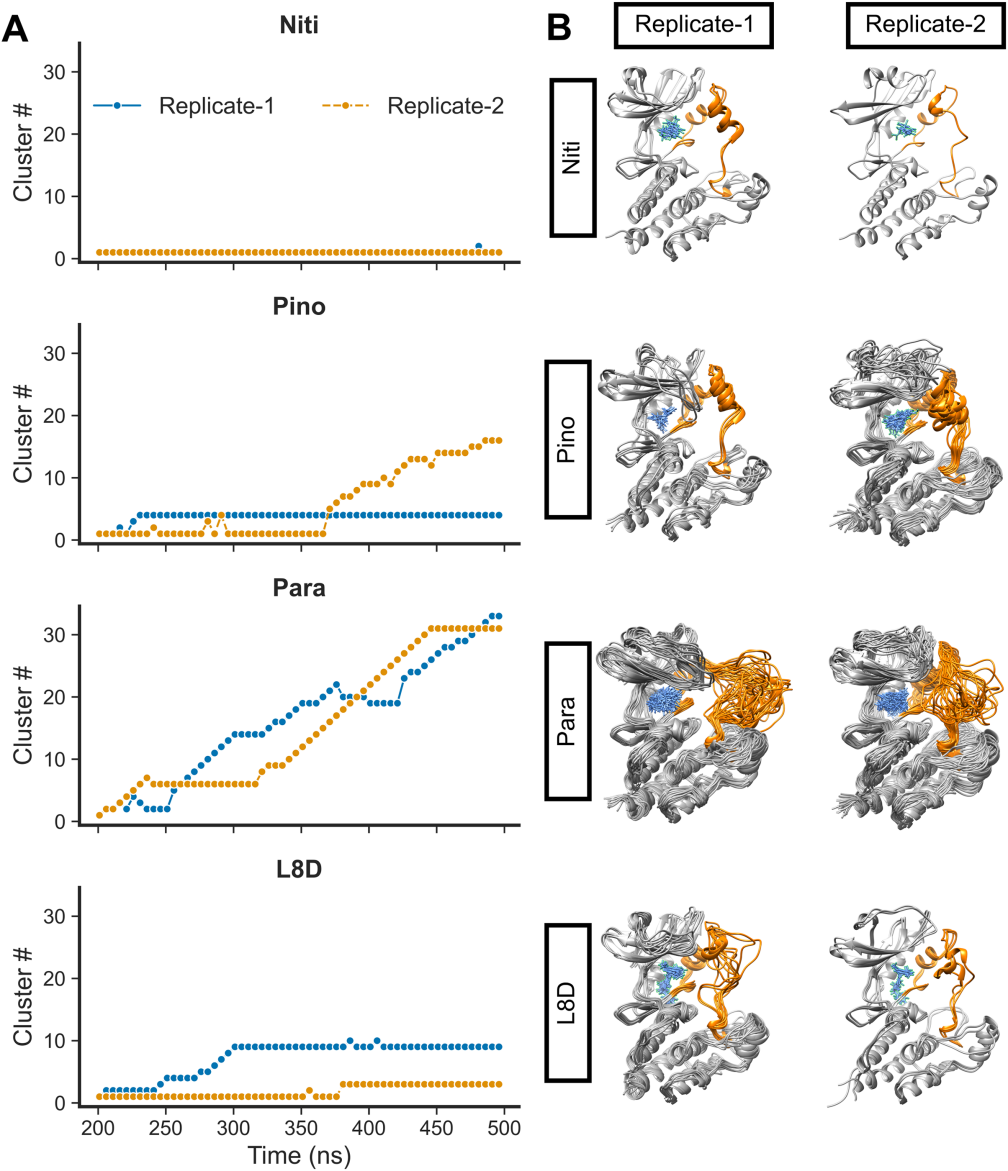
RMSD clusters of protein-ligand complexes over time. (A) Time *vs*. cluster IDs. (B) Average 3D structures of the complexes from clusters.

We then calculated the clustering of RIPK1 complexes by evaluating the RMSD values (Fig. 4A). Structures were grouped if their RMSD values did not exceed 0.15 nm. Additionally, we derived the average 3D structures for the clusters (Fig. 4B). Notably, the RIPK1-Nitiducarpin exhibited a high degree of stability, displaying predominantly a single conformation throughout the entire simulation period (Fig. 4). For the reference ligand L8D, we identified ten and three clusters in Replicate-1 and Replicate-2, respectively. In one replication, the RIPK1 complex with the ligand Pinocembrin 7-O-benzoate underwent a few initial conformational changes and then stabilized at the 4^th^ cluster for the remainder of the simulation. The same complex transitioned through 16 clusters in another replication, with rapid conformational changes occurring predominantly within the last 140 ns. Conversely, the Paratocarpin J complex did not appear to converge. Instead, it formed a rapidly evolving series of protein-ligand conformations throughout the simulation period, as illustrated in Fig. 4. Remarkably, conformational changes were evident primarily at the activation loop of RIPK1 (residues 156–196) (Fig. 4B, orange).

**Figure 5:**
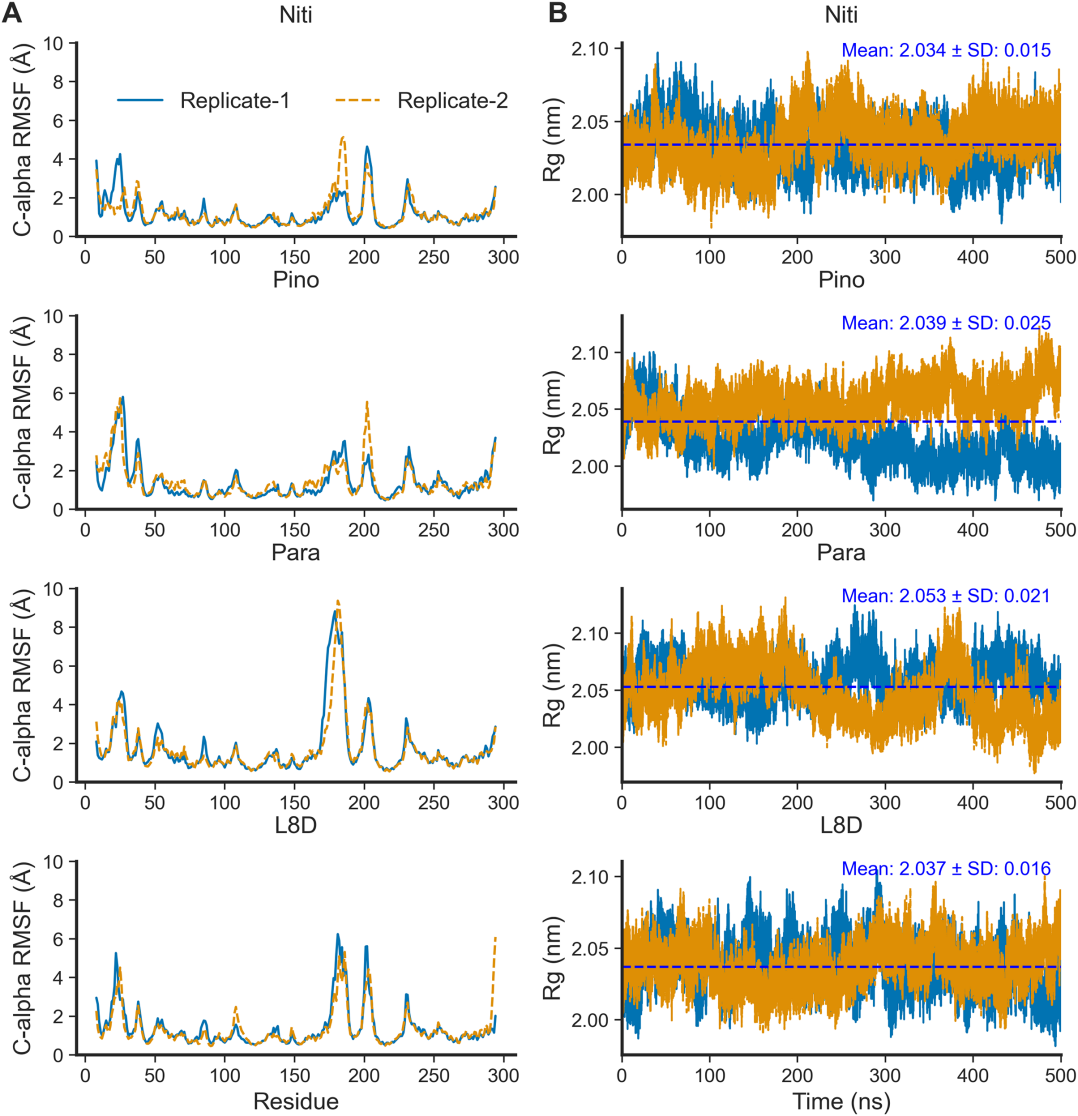
Local fluctuation of the protein residues and global changes of the protein compactness. (A) Protein C-alpha RMSF. (B) Protein Rg.

For the top complexes, we estimated local fluctuations of the RIPK1 C-alpha atoms by evaluating the per residue RMSF values (Fig. 5A). Generally, loose ends and loop regions of proteins undergo higher conformational changes. The RIPK1 P-loop corresponding to the residues 24–31 displayed RMSF values in the range of 4 to 6 Å for all complexes. However, the maximum conformational rearrangements were evident for the activation loop residues (amino acids 156–196), the Paratocarpin J complex generating the highest peaks in both replicates (Fig. 5A). Residues (amino acids 198–208) adjacent to the activation loop also showed higher fluctuations, although there were no remarkable differences among the top complexes.

The compactness of a protein, often estimated by the Rg from MD simulation trajectories, infers protein stability in a biological system. A lower Rg value suggests more compactness and increased protein stability. For the top ligands, the mean Rg values ranged from the minimum of 2.034 nm for Nitiducarpin to the maximum of 2.053 nm for Paratocarpin J (Fig. 5B). Notably, the standard deviation was also the lowest (±0.015 nm) for RIPK1 complexed with Nitiducarpin, denoting a more stable binding of this ligand.

**Figure 6:**
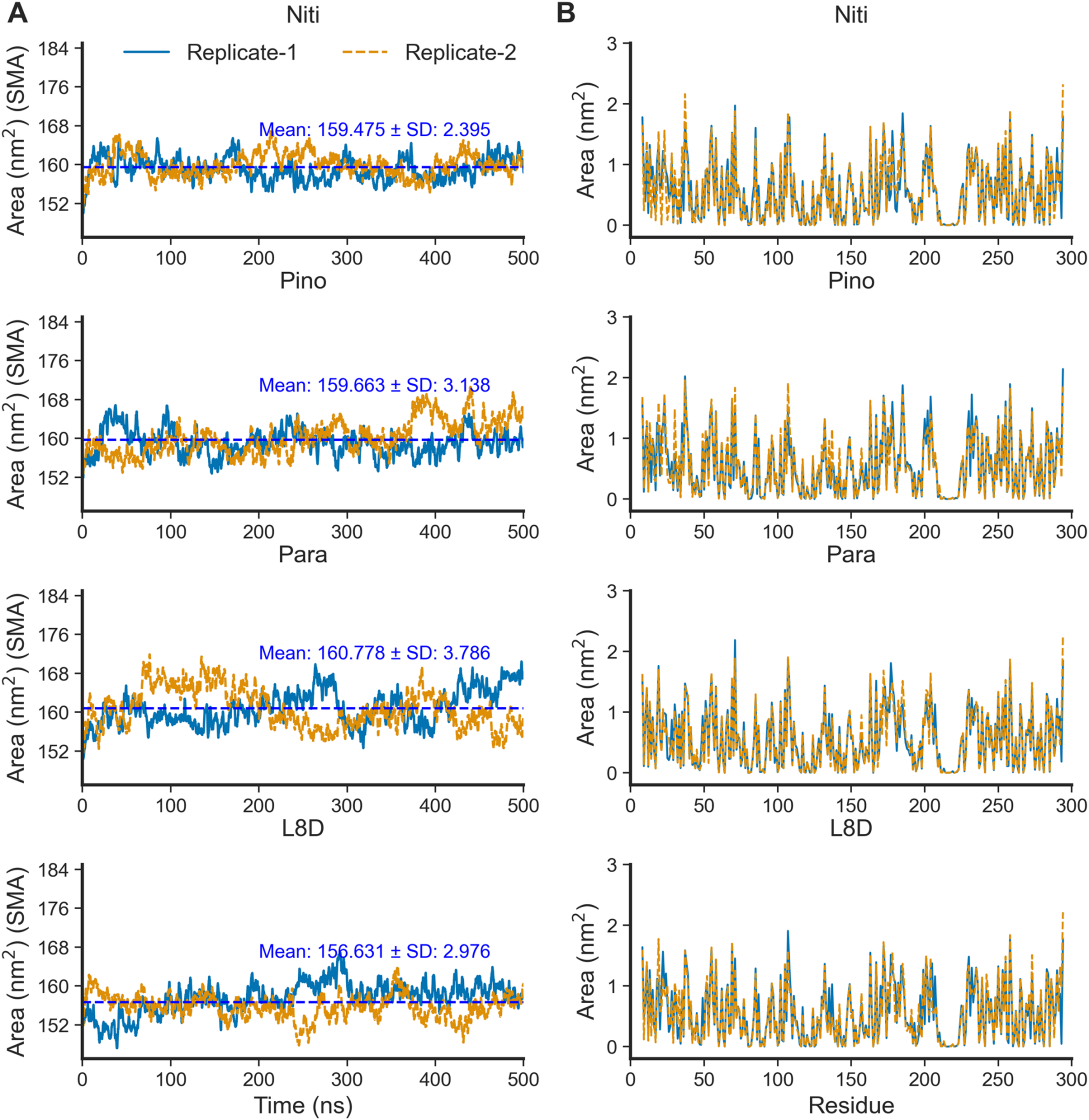
SASA of RIPK1 complexed with the top ligands. (A) SASA over time. (B) SASA per residue. SMA: simple moving average of 100 data points.

The SASA of proteins is a robust estimator of protein folding and stability and has been employed in predicting protein-ligand binding and pathogenicity of protein mutations (Núñez, Venhorst & Kruse, 2010; Wei, Brooks & Frank, 2017; Savojardo et al., 2021). In MD simulations of protein-ligand complexes, a significant increase in SASA can be translated into a departure of the ligand from the binding pocket. We calculated protein SASA values from the trajectories using the gmx sasa tool (Fig. 6). Interestingly, RIPK1 complexed with Nitiducarpin exhibited the most stable SASA values (Mean: 159.475 nm^2^, SD: ±2.395 nm^2^) while the variation was maximum for Paratocarpin J (Fig. 6A). Nevertheless, per residue SASA values did not display any remarkable changes among the complexes (Fig. 6B).

**Figure 7:**
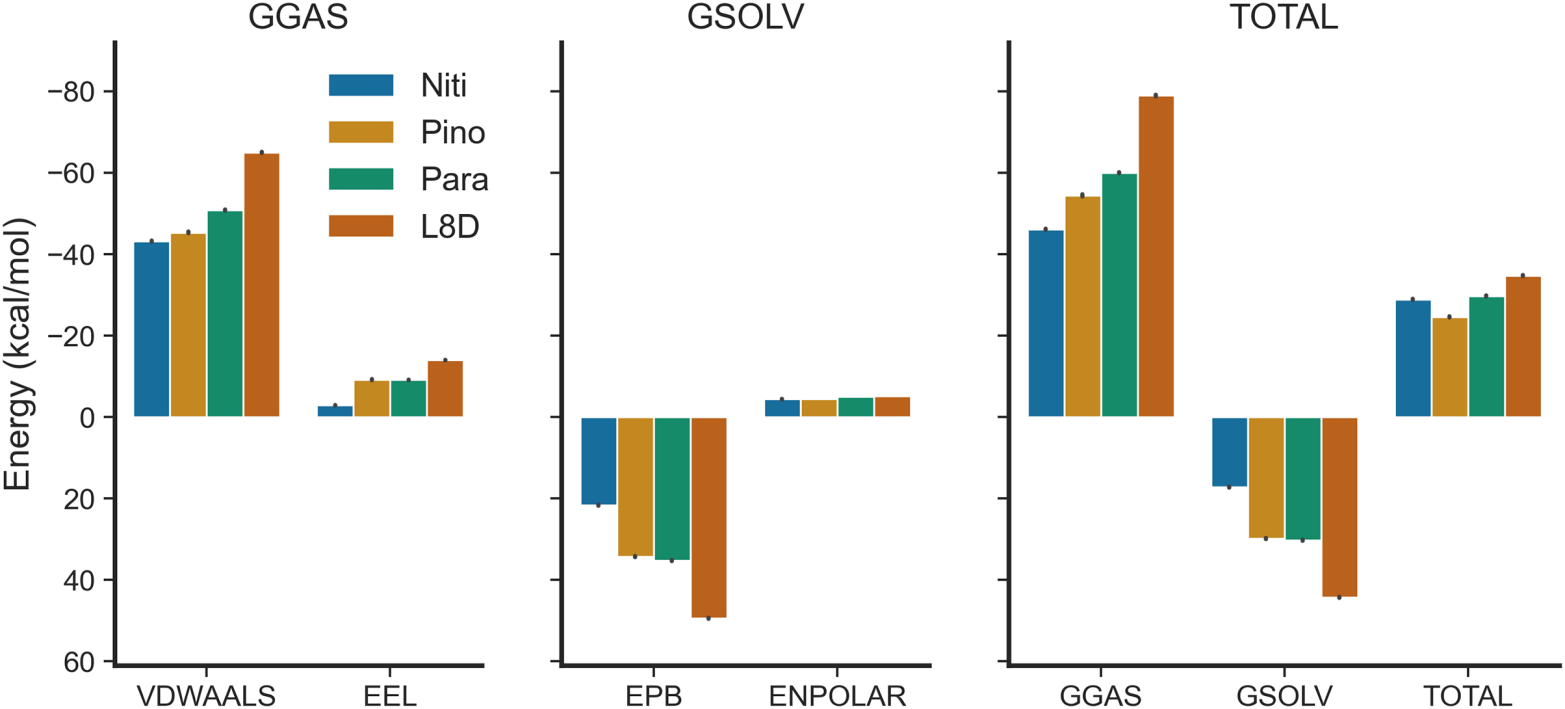
Binding free energy of the top ligands calculated using the MMPBSA method.

We estimated the binding free energies of the top ligands using the MMPBSA method (Fig. 7). The gas phase energy (GGAS) was contributed mainly by van der Waals interactions (VDWAALS) and electrostatic interactions (EEL). The reference ligand L8D displayed the strongest VDWAALS and EEL. However, the differences among the top three ligands were not substantial (Fig. 7). All ligands showed small and indistinguishable ENPOLAR values. Variations in GSOLV values were influenced by EPB values, with Nitiducarpin having the lowest GSOLV and L8D exhibiting the highest (Fig. 7). Total enthalpies were −28.857, −24.624, −29.699, and −34.741 kcal/mol for Nitiducarpin, Pinocembrin 7-O-benzoate, Paratocarpin J, and L8D, respectively (Fig. 7). To decipher the roles of binding residues, we also analyzed per residue total energy decomposition using the MMPBSA method (Table 2). Residues within 4 Å of the ligand were selected for analysis. Val31, Ile43, Met92, Ala155, Leu157, and Leu159 were found to play roles in ligand binding. Notably, Met92 and Leu157 made the most significant contributions (Table 2).

**Table 2:** Per residue total energy decomposition using the MMPBSA method.

Ligand	Replicate	Total energy decomposition (kcal/mol)					
		Val31	Ile43	Met92	Ala155	Leu157	Leu159
Niti	Replicate-1	−1.27	−0.73	−1.96	–	−2.02	−1.02
Niti	Replicate-2	−0.95	–	−1.9	–	−2.03	−1.06
Pino	Replicate-1	−0.63	−1.01	−2.41	−1.48	–	−0.78
Pino	Replicate-2	−0.78	−1.16	−1.61	−1.51	−1.18	–
Para	Replicate-1	−1.27	−0.86	−1.7	−0.57	−0.89	−0.81
Para	Replicate-2	−1.06	−0.72	−2	−0.85	−1.43	−1.19
L8D	Replicate-1	−0.57	–	−2.36	−1.25	−1.73	−0.99
L8D	Replicate-2	−0.79	−0.58	−2.58	−1.14	−1.48	−0.9
L8D	Replicate-2	−0.79	−0.58	−2.58	−1.14	−1.48	−0.9

**Figure 8:**
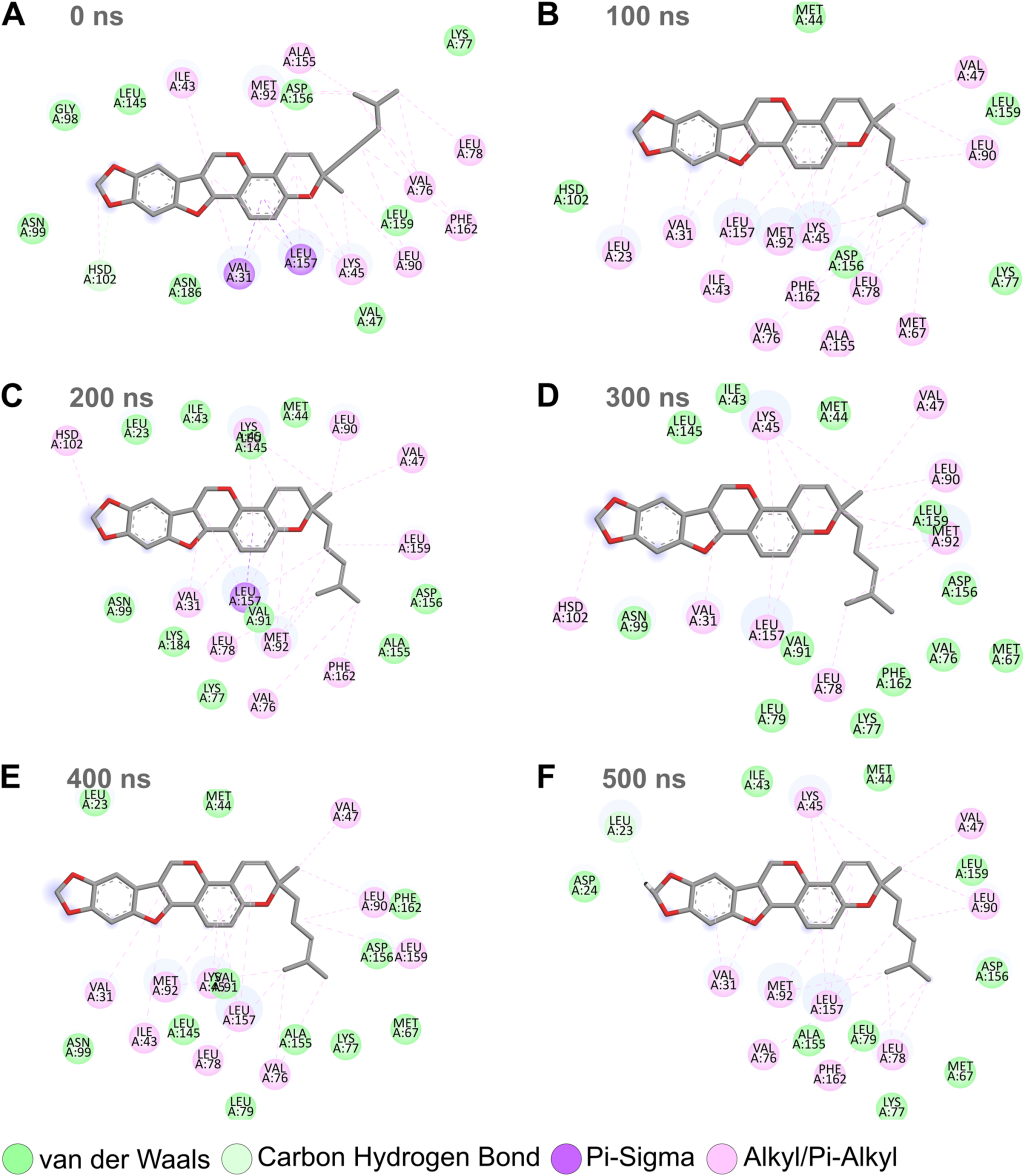
Protein-ligand binding interactions of Nitiducarpin and RIPK1. Snapshots were retrieved at 0 ns (A), 100 ns (B), 200 ns (C), 300 ns (D), 400 ns (E), and 500 ns (F) from the simulation trajectory.

Combining the above RMSD, RMSF, and SASA analyses for the MD simulation trajectories of the top protein-ligand complexes (Figs. 3–6), Nitiducarpin demonstrated the most favorable binding to RIPK1. To further reveal the stability of the RIPK1-Nitiducarpin complex, we generated 2D protein-ligand interaction diagrams from snapshots retrieved from the simulation trajectory at 100 ns intervals (Fig. 8). We observed a clear predominance of nonpolar interactions of Nitiducarpin (Fig. 8), as expected from the target hydrophobic binding cavity in our molecular docking step. A stable ligand binding was governed primarily through VDWAALS and Alkyl/Pi-Alkyl interactions (Fig. 8).

Similar to canonical kinases, the kinase activity of RIPK1 largely depends on the catalytic triad residues Lys45, Glu63, and Asp156, P-loop residues 24–31, and the catalytic loop residues 136–143 (Xie et al., 2013). We observed that Nitiducarpin formed stable Alkyl/Pi-Alkyl interaction with Lys45 and VDWAALS with Asp156, the two residues of the kinase catalytic triads (Fig. 8). An interaction with the P-loop residue Val31 was also evident at all time points. Other significant interacting residues include Met67, Val76, Leu78, Leu90, Met92, Leu157, and Phe162 (Fig. 8). Overall, Nitiducarpin is predicted to interact with many key residues governing a stable binding of the reference ligand L8D to RIPK1 (Fig. 8) (Xie et al., 2013).

**Figure 9:**
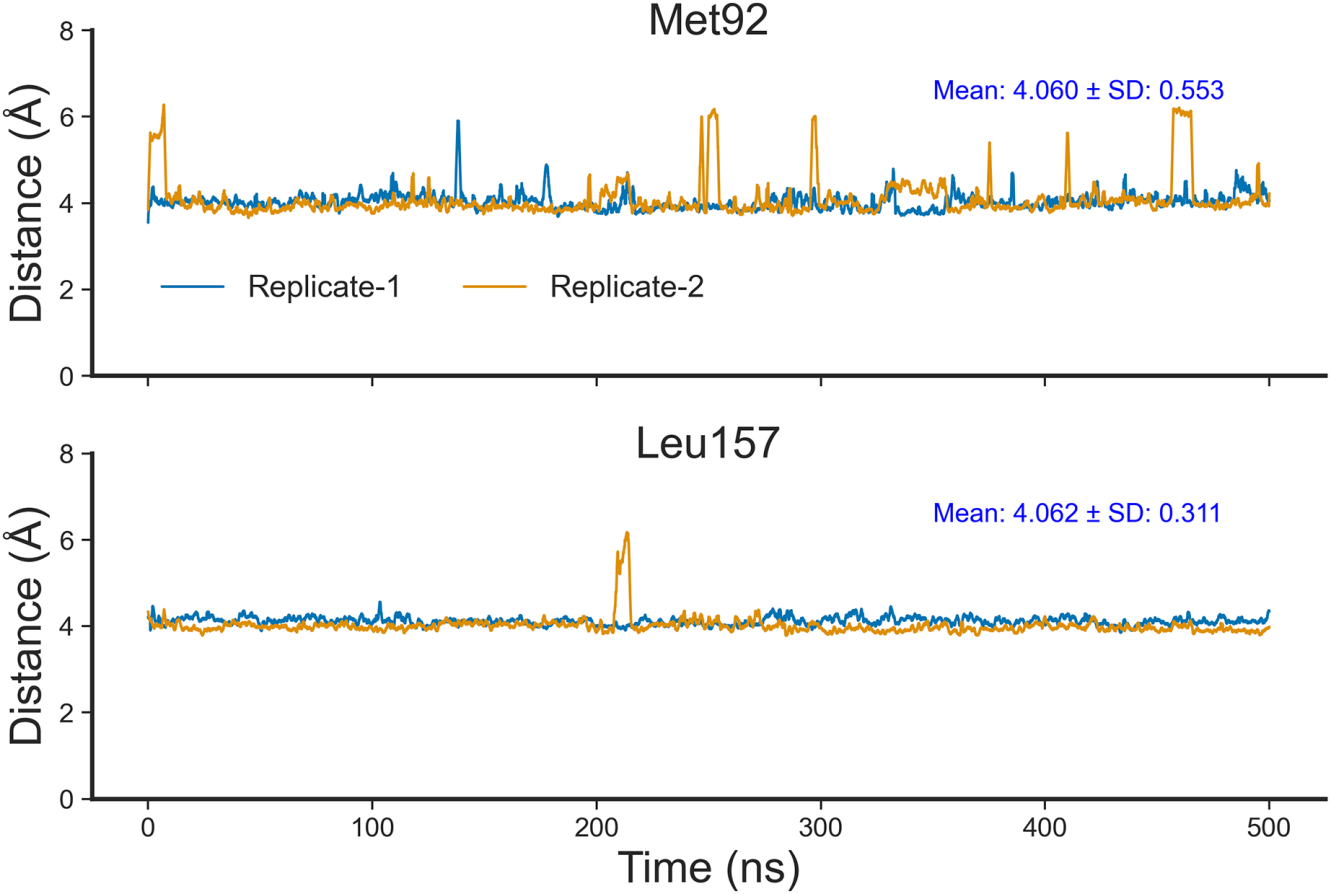
Distances of Nitiducarpin from the residues Met92 and Leu157.

To confirm that Nitiducarpin could indeed form stable interactions with RIPK1, we calculated the distances of the two key residues, Met92 and Leu157, from the ligand (Fig. 9). The analysis revealed that Nitiducarpin stayed within a mean distance of 4.060 Å and 4.062 Å from Met92, and Leu157, respectively. We observed brief departures from Met92 on several occasions. Nevertheless, stable interactions were maintained with Leu157 throughout the simulation period (Fig. 9).

In summary, we screened 4,858 flavonoids using molecular docking simulations. We shortlisted 20 ligands based on binding affinities, which were then subjected to ADME screening, returning three orally bioavailable and blood-brain barrier penetrant compounds: Nitiducarpin, Pinocembrin 7-O-benzoate, and Paratocarpin J. We validated binding interactions using replicated molecular dynamics simulations, compared the results with the strong RIPK1 inhibitor L8D, and identified Nitiducarpin as the most potent ligand for RIPK1. To be noted, Nitiducarpin (PubChem CID: 630656) is an isoflavonoid of the pterocarpan subclass with the chemical formula C_26_H_26_O_5_ and a molecular weight of 418.48 g/mol.

## Discussion

In the present study, we employed computational tools to sequentially screen a library of 4,858 flavonoids using molecular docking, ADME analysis, molecular dynamics simulations, and free energy calculations. Our virtual screen yielded three flavonoids as promising RIPK1 inhibitors predicted to be bioavailable and CNS penetrant.

Recent preclinical and clinical studies have unquestionably recognized the roles of RIPK1 in neurogenerative disorders (Li & Yuan, 2023; Scarpellini et al., 2023; Xu, Zhang & Zhuang, 2023). In a phase I clinical trial, a small molecule GFH312 showed significant RIPK1 inhibition with a tolerability profile comparable to that of the placebo (Lickliter et al., 2023). Despite demonstrating promise in targeting RIPK1 for the treatment of Alzheimer’s disease, SAR443060, another orally bioavailable CNS penetrant investigational agent, was abandoned due to long-term toxicities (Vissers et al., 2022). A reversible RIPK1 inhibitor, GDC-8264, successfully attenuated RIPK1 activation in healthy volunteers with only mild adverse effects, supporting the development of therapies targeting RIPK1 (Jones et al., 2023).

Flavonoids are being explored increasingly in modulating the RIPK1/RIPK3/MLKL pathway. Morin, a plan-derived flavonol, was able to prevent cognitive deficits, necroptosis, and mitochondrial damage in a rat model of neurodegeneration, apparently through Ripk1 inhibition (Abd El-Aal, El-Abhar & Abulfadl, 2022). Luteolin, a flavone known for antineoplastic effects, was also found to resist dexamethasone-induced activation of RIPK1 and necroptosis in primary human bone microvascular endothelial cell cultures (Xu et al., 2023).

After validating the three hits from molecular docking studies using long replicated molecular dynamics simulations and free energy calculations, Nitiducarpin, an isoflavonoid plant metabolite, displayed the most stable binding interactions with the target RIPK1. Low RMSD values with minimal deviations and quick convergence in MD simulations implied that Nitiducarpin can settle at the target’s hydrophobic binding cavity, forming a stable protein-ligand complex.

Previous studies confirmed that potent RIPK1 inhibitors interact with the residues that play critical roles in the kinase activity (Xie et al., 2013). A recent study on the inhibition of RIPK1 also revealed that the amino acid residues Asp156 and Lys45 are critical to ligand binding (Zhang et al., 2023). Our analysis demonstrated stable hydrophobic interactions of Nitiducarpin with many crucial amino acid residues, including Val31, Lys45, Met92, Asp156, Leu157, and Phe162.

We identified Nitiducarpin, a plant-derived flavonoid, as a potent RIPK1 inhibitor through comprehensive computational studies. This compound could serve as a potential lead in future *in vitro* and *in vivo* experiments aiming at discovering therapeutics for neurodegenerative diseases, including AD, PD, and HD.

## Conclusions

Aberrant regulations of the kinase RIPK1 play pivotal roles in neuroinflammation and necroptosis, contributing to the pathophysiological cascades in neurodegeneration, making RIPK1 a prime target for CNS drug discoveries. We virtually screened a chemical space of 4,858 flavonoids using molecular docking, pharmacokinetic evaluation, and molecular dynamics simulations to discover potent RIPK1 inhibitors with optimum bioavailability and blood-brain barrier permeability. Among the three hits from docking and ADME screening, Nitiducarpin qualified to be a strong inhibitor possessing favorable physicochemical properties and capable of forming stable protein-ligand interactions with RIPK1. Our discovery holds promise for advancing the development of RIPK1 inhibitors for treating neurodegenerative disorders.

## Supplemental Information

10.7717/peerj.16762/supp-1 Supplemental Information 1 Equilibration and emergy minimization of the MD simulation systems.

10.7717/peerj.16762/supp-2 Supplemental Information 2 Pharmacokinetic properties of top 20 ligands predicted from SwissADME.

10.7717/peerj.16762/supp-3 Supplemental Information 3 The flavonoid library used in the screen.

10.7717/peerj.16762/supp-4 Supplemental Information 4 Topology files for MD simulations for the best ligand Nitiducarpin.

10.7717/peerj.16762/supp-5 Supplemental Information 5 Initial and final pdb files for the Nitiducarpin-RIPK1 complex for both replicates.

## Additional Information and Declarations

### Data Availability

The following information was supplied regarding data availability: The ADME profiles are available in the Supplemental File.
