# Supplementary figures and images for: Virtual screening of flavonoids as potential RIPK1 inhibitors for neurodegeneration therapy
Source: PeerJ. 2024 Jan 22;12:e16762. doi: 10.7717/peerj.16762 (PMC10809995; doi:10.7717/peerj.16762)

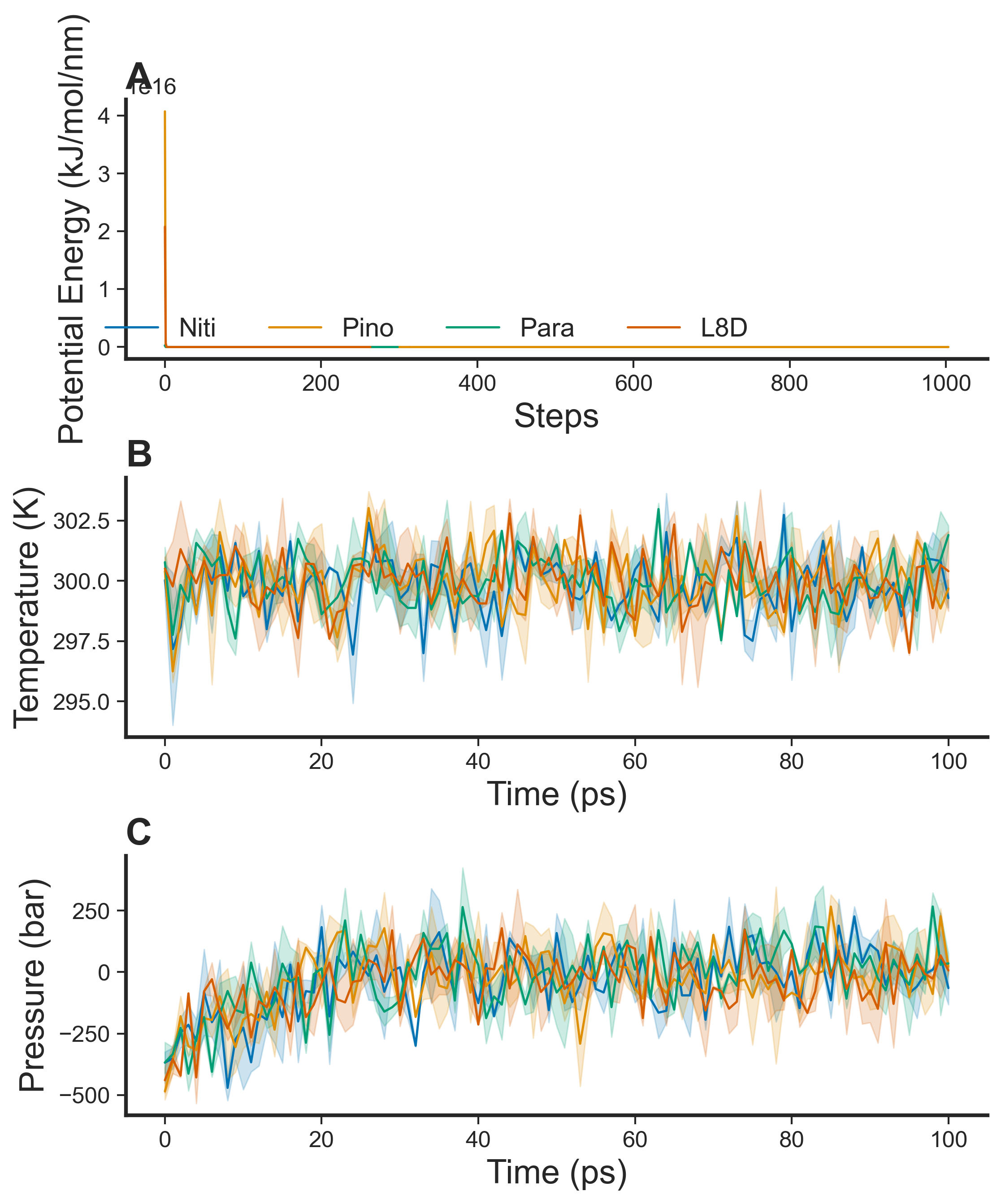

Supplement: Supplemental Information 1 [file peerj-12-16762-s001.png]
